# Justification of Starching Cotton and Aramid Yarns by Industrial and Laboratory Processes

**DOI:** 10.3390/polym15112448

**Published:** 2023-05-25

**Authors:** Ana Kiš, Ivana Schwarz, Ružica Brunšek, Stana Kovačević

**Affiliations:** 1 Vertiv Croatia Ltd., Oreškovićeva ulica 6n/2, 10010 Zagreb, Croatia; akis@ttf.unizg.hr; 2 Department of Textile Design and Management, Faculty of Textile Technology Zagreb, University of Zagreb, Prilaz baruna Filipovića 28a, 10000 Zagreb, Croatia; stana.kovacevic@ttf.unizg.hr; 3 Fibres and Textile Testing, Department of Materials, Faculty of Textile Technology, University of Zagreb, Prilaz baruna Filipovića 28a, 10000 Zagreb, Croatia; ruzica.brunsek@ttf.unizg.hr

**Keywords:** aramid yarn, cotton yarn, yarn starching, industrial starching, laboratory starching, tensile properties, abrasion resistance

## Abstract

The development and application of new types of fibres and their wider application influence the continuous invention of a more economical starching process, as one of the most expensive stages in the technological process of woven fabric production. For example, aramid fibres are increasingly used in clothing with effective protection from mechanical, thermal and abrasion exposure. Simultaneously, comfort and regulation of metabolic heat are extremely important, and this is achieved by using cotton woven fabrics. For such a woven fabrics to satisfy the protective properties and the possibility of all-day wear, fibre is needed, and thus a yarn, that will enable the efficient production of fine, light and comfortable protective woven fabrics. This paper investigates the influence of starching on the mechanical properties of aramid yarns and their comparison to cotton yarns of the same fineness. This will lead to knowledge about the efficiency and necessity of aramid yarn starching. The tests were carried out on an industrial and laboratory starching machine. According to the obtained results, the necessity and the improvement of the physical-mechanical properties of cotton and aramid yarns can be determined, both by industrial and laboratory starching. Finer yarn starched by the laboratory starching process achieves greater efficiency in the yarn’s strength and resistance to wear, which indicates the need for starching aramid yarns, especially fineness 16.6 × 2 tex, but also finer ones.

## 1. Introduction

Every innovation in the starching process of spun yarns, which brings a new approach, new methods or optimizes the existing ones, represents a contribution to the production of woven fabrics. The aim of starching is to improve the properties of the yarn before the weaving process, if the yarn does not have satisfactory strength, sufficient resistance to wear and generates static electricity during the processing. The fibre and yarn processing stages before the starching process, as well as the processes that follow starching, affect the final quality and economic justification of starching. The properties of most spun yarns are not at a level that would meet the target quality and optimal woven fabric production. In order to realize the weaving process with a satisfactory utilization of the weaving machines and a satisfactory quality of the produced fabric, the warp and weft yarns must have certain properties that ensure the set goals [1,2]. Starching makes it possible to achieve the necessary properties that guarantee the target quality of the finished product. The efficiency of starching depends not only on the adhesion between the applied starching agent and the yarn but also on the ability to form a surface layer of starch pick-up, the rheological properties of the starch mass, the physico-chemical properties of the yarn, as well as on the technological parameters of the starching process itself [3,4].

The starching process is a common stage in woven fabric production, but not a necessary one. Some yarns have satisfactory physico-mechanical properties and resistance to long-term cyclic stresses, so it is not necessary to starch them. However, in order to achieve efficient production and the quality of the finished product, it is often necessary to protect the yarn from both internal and surface damage caused by strong and two-way abrasion against the metal elements of the machine during the weaving process, at the time of its pronounced tension, despite its relatively high strength. Since starching is an extremely expensive stage in woven fabric production, it has always been one of the main factors in achieving a more economical and high-quality production [5,6,7,8,9].

The stages of fibre and yarn processing before starching, as well as those that follow until the moment of production of the finished woven fabric, affect the final quality and economic justification of starching. Most of the spun yarns, with their key properties, do not allow to meet the quality and optimal fabric production, and therefore in such cases, it is necessary to implement the starching process. This increases the yarn strength and abrasion resistance and realizes the weaving process with a satisfactory utilization of the weaving machines and a satisfactory quality of the produced woven fabric [10,11].

Fibres spun into yarn are often of insufficient strength and uneven, especially cotton fibres, so they usually need to be starched. By applying appropriate starch agents and their share in the starch mass, it is possible to achieve effective protection of the warp threads [12]. Each warp type requires a special approach in starching, because each yarn (although of the same raw material composition and the same characteristics) has differences and deviations in properties (for example, cotton from different climates, different spinning mills, etc.) [13]. For this reason, it is not possible to standardize the starching conditions, which emphasizes the complexity of the starching process itself. The continuous development of even finer synthetic fibres provides the possibility of spinning extremely fine threads, which is followed by the production of ever denser and finer fabrics. This kind of production of woven fabrics with maximum densities of extremely fine warp threads requires extreme caution. Furthermore, with increasingly improved and innovative starching agents and expertise in managing starching processes, it is possible to achieve maximum effects of the starching process [14,15,16,17,18]. Despite high technological progress and the development of new, more effective starch agents, there is always room for improvement in this process stage. If we take into account the development of new textile raw materials and their processing from fibre to finished fabric, starching remains a continuous focus of monitoring the entire process.

Today’s modern starching plants with full automation of the starching process enable systematic control and regulation of key parameters. However, despite this, as well as the good properties of cotton and aramid yarns, caution is still needed in fabric production processes, especially when using finer fibres and yarns, which are increasingly used to produce fabrics for light and comfortable clothing, with simultaneous properties of protection from high temperatures and mechanical impacts.

Analyzing starch from the physico-chemical side, there are still problems in achieving the optimal effect of the starching process, which are manifested in [19]:

- the size of the molecules that limits their penetration into the spaces between the fibres in the yarn,
- unstable viscosity of the starch mass during temperature changes,
- increasing the stiffness, or decreasing the elasticity of the yarn,
- possible sensitivity to rotting and degradation by microorganisms.

With this research, an attempt is to find the justification and importance of starching yarns of different raw material compositions, different finenesses, and thus different properties, in a laboratory as well as in industrial conditions. The yarns raw materials compositions that were used for the implementation of this research are cotton and aramid, for the reason that both types of yarn are largely represented in the production of protective woven fabrics for protective clothing. Their protective properties determine their position in relation to the body, where aramid is present on the outer layer, effectively protecting the body from mechanical, thermal and abrasion exposure; while cotton is present on the inner layer, ensuring comfort and regulation of metabolic heat.

### Starching of Cotton and Aramid Yarns

**Figure 1:**
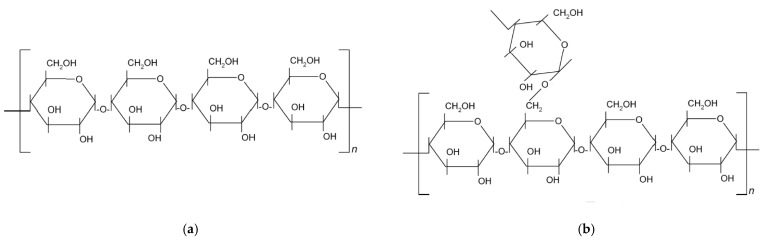
Chemical structure of starch with (**a**) amylose and (**b**) amylopectin units.

Starch is a very important and widely distributed natural product, occurring in the leaves of green plants, seeds, fruits, stems, roots, and tubers. Chemically, starch is composed of two kinds of polysaccharides, amylose (semicrystalline biopolymer soluble in hot water) and amylopectin (highly crystalline, insoluble in hot water) consisting of a large number of glucose units joined by glycosidic bonds (Figure 1). Amylose has a linear structure that provides the crystalline structure of the starch, and amylopectin, has a branched structure that presents the amorphous phase of the starch [20,21,22]. Depending on the source, starch generally contains amylose and amylopectin in a ratio of 15/85% to 30/70%. In both components, there are primary and secondary hydroxyl groups in every glucose unit, with different reactivity [20,23].

Starch is the basic agent for yarn starching in the process of woven fabric production to protect against dynamic stresses and two-way friction in the weaving process. By starching, an even layer of protective coating is formed on the yarn and the laying (smoothing) of protruding fibres along the body of the yarn. Other important components that are dosed into the starch mass besides starch are glues, fats, antimicrobial and antistatic agents, cross-linking agents, etc. The proportion of individual components depends on the type and properties of the yarn, the production technology, the type of weaving machine and the parameters of the designed fabric. In order to keep the fibres in the yarn during the weaving process in the position they were in before starching and to make the yarn smooth, it is necessary to bond the starch with the fibres in the yarn to such an extent that the yarn is preserved from destruction, but also that the removal of starch after the weaving process (destarching) be simple. Optimizing the adhesion strength of starch to fibres and the ease of removal in the destarching process is in a constant focus on starching improvement and the development of new agents [24].

Cotton fibre is the purest source of cellulose and the most significant natural raw material for the production of textiles for various areas of use. It is widely used due to its excellent wearability, superior wearing comfort and excellent moisture permeability [25]. Cotton fibre is a natural cellulosic fibre with a biologically complex structure, but chemically it is mainly composed of cellulose. In addition to cellulose, other natural non-cellulosic substances are also present in smaller quantities in the fibre such as pectins and waxes that are the most responsible for the hydrophobicity or low water wettability of raw cotton fibres. Along with waxes and fats, cotton fibre also contains proteins and minerals, and they are mostly found in the outer part of the fibre [25,26]. As the basic building material with the highest structural order among all plant fibres (highly crystalline, oriented and fibrillary), cellulose significantly determines the chemical and physical properties of cotton fibres.

**Figure 2:**
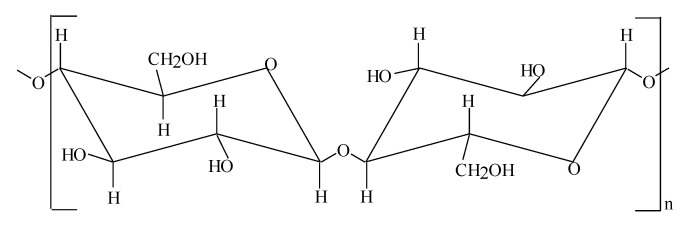
Chemical structure of cellulose.

Cellulose is a polysaccharide whose molecule consists of a linear series of units of β-D-glucose residues connected by strong covalent glucosidic bonds between the first and fourth C-atoms (1–4 bonds). Every other anhydroglucose β-D-glucose residue in the molecule is rotated by 180° with respect to the previous one. Such a syndiotactic arrangement contributes to the fact that the three active hydroxyl groups of one anhydroglucose residue, one primary alcoholic and two secondaries, are always in the opposite position in relation to the polymeric chain (Figure 2). In addition, the structure of the macromolecule is represented by the repetition of cellobiose [25,26,27], where the rings are slightly bent in the shape of a saddle, respectively a saddle conformation.

Aramid fibres are special polyamide fibres, which are spun from solutions of aromatic polyamides. In at least 85% of amide or imide bonds in the macromolecule, they directly connect benzene rings. According to the basic structure of macromolecules, two types are distinguished:

- m-aramid fibres—in which amide and imide groups connect aromatic constitutional units in meta-position (Nomex, Durette, Conex, Apyeil),
- p-aramid fibres in which amide and imide bonds are in para-position (Kevlar, Technora, Tawron, Kermel).

**Figure 3:**
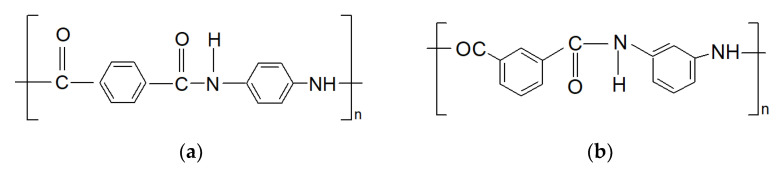
Chemical structure of (**a**) p-aramid, (**b**) m-aramid.

The difference between these two types is in the position of the bonds on the carbon atoms in the ring: meta-aramid has bonds in positions 1 and 3 (distant by two atoms), while para-aramid has bonds in positions 1 and 4 (diametrically opposite) [26,28] (Figure 3).

Due to their exceptional thermal resistance, aramid fibres do not lose their strength properties at elevated temperatures (up to 200 °C), they have good mechanical properties under load, high limiting oxygen index, excellent mechanical and chemical stability, antistatic properties, and they are good heat insulators, they are not prone to peeling, and are easy to maintain. They have weaker compressive properties and resistance to UV radiation and have a low density (ρ~1.45 g/cm^3^). Aramid fibres are highly anisotropic and susceptible to degradation with strong acids and alkalis but are relatively inert to other solutions and chemicals [29,30].

Starching of aramid yarns, which are increasingly used for light and comfortable clothing, simultaneously serves to protect against high temperatures and mechanical shocks. Despite the good properties of aramid fibres, a kind of caution is required when using them in woven fabric production processes, especially finer fibres and yarns. Their extraordinary properties enable them to be used in a wide range of applications. In order for the fibres, then the yarn and the woven fabric, to maintain strength and elasticity, it is extremely important to determine the right technological processing. Starching is an important processing stage that directly affects the properties of the final product. The morphological and chemical structure of aramid fibres, as well as the method of yarn production (multifilament or cut filaments and spun into yarn), affects the bonding method to starch and the quality of starching. Macromolecules of aramid fibres, where the aromatic rings are connected by amide bonds (-CO-NH-), enable connections with starch. Furthermore, aramid fibres are circular in cross-section and have a smooth surface compared to cotton fibres which are irregular in cross-section with a non-uniform surface. Due to the aforementioned morphological difference, aramid fibres provide a denser, more compact and uniform yarn than cotton fibres, with less air space inside the fibre, whereby the starch mass fills the empty spaces inside the aramid yarns more difficult. Given that cotton yarn, due to its uneven morphology, has larger air spaces inside the yarn, the penetration of the starch mass into the interior of the yarn is easier and more uniform.

If aramid fibres are cut and twisted into yarn, air spaces are created inside the yarn, and a larger amount of starch, which does not chemically bond with the fibres, remains trapped in the interstices of the fibres and a protective film is formed on the surface.

The stickiness of the starch mass allows it to be connected and retained in the yarn, even after the water removal, i.e., after drying. Since the removal of starch is necessary after weaving, it is important that its removal from the yarn is as easy as possible, which again emphasizes the importance of optimizing the starch pick-up in the starching process [31].

To get the most accurate answer to the question of whether a particular yarn should be starched and to what extent, a detailed analysis of the yarn and starching agents, the target properties of the finished fabric, the available starching facilities, and the existing conditions of the production process, is required. According to the available literature, it is evident that the area of aramid yarns’ starching has not been sufficiently researched so that we could say with certainty whether aramid yarns need to be starched or not. Despite the high technological progress and the development of new, more efficient starch agents, there is always an opportunity to improve this process phase. Taking into account the development of new textile raw materials and their processing from fibre to finished woven fabric, starching becomes and remains continuously a focus of process monitoring.

## 2. Materials and Methods

### 2.1. Yarns

Two types of yarn produced in Litia Spinnery, Slovenia, were used in this paper: 100% cotton and 95% meta-aramid + 5% para-aramid, in 4 fineness’s, which have been subjected to industrial and laboratory starching. The fineness of the tested yarn samples is expressed according to the international units system in Tex. Yarn samples were divided into 8 groups, according to fineness and raw material composition (Table 1). The samples are marked according to the type of yarn raw material composition (C—cotton, A—aramid) and yarn fineness (expressed in Nm).

### 2.2. Starch Agent

The starching agents used in this research were the following: Fibrosint C75 (Pulcra Chemicals GmbH), Inex 773C (Pulcra Chemicals GmbH), Avirol 308AS (Pulcra Chemicals GmbH). Their share in the starch mass, which were identical in both starching processes (industrial and laboratory) are shown in Table 2.

### 2.3. Industrial and Laboratory Starching

Industrial starching was carried out in the company Čateks Ltd., Čakovec, Croatia, at the starching plant tt. Polmatex Wolma, model A4, Poland. Starch agents, their share in starch mass, cooking temperature and time, and concentration of starch mass were identical in the industrial and laboratory starching process.

**Figure 4:**
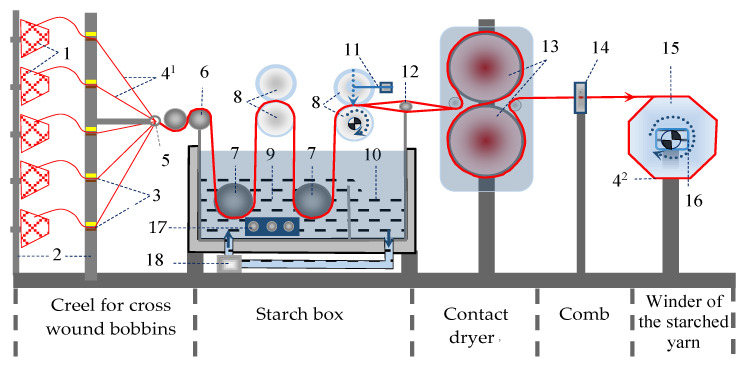
Laboratory starching device: 1- cross wound bobbins, 2—creel for cross wound bobbins, 3—thread guides and tensioners, 4^1^—unstarched yarn, 4^2^—starched yarn, 5—thread guide, eyelet, 6—rollers for thread guidance, 7—rollers for dipping the yarn in starch mass, 8—rollers for squeezing out excess starch mass, 9—starch mass in the starch box, 10—starch mass in the reserve box, 11—regulation of the force of extrusion, i.e., regulation of starch pick-up, 12—roller for wet division of yarn, 13—rollers for contact yarn drying, 14—comb for separation of dry yarn, 15—winch for winding starched yarn, 16—electric motor with speed regulation, 17—switches for: driving the last pair of rollers for squeezing out the starch mass, turning on the pump and turning on the starch mass heater with by temperature regulation, 18—pump for the circulation of starch mass from the starch box to the reserve box.

Laboratory starching was performed on the starching device shown in Figure 4. The laboratory starching device was constructed at the University of Zagreb Faculty of Textile Technology (recognized patents under the numbers PK20070247 and PK20070248 at the State Institute for Intellectual Property of the Republic of Croatia).

The laboratory conditions of starching were adapted to industrial conditions, and are shown in Table 3. Starching was carried out on 3 threads per type of yarn, i.e., a total of 24 threads for 8 samples. The total length of the starched yarn was 200 m, of which 40 m from the beginning of starching and 10 m from the end of starching were discarded, so the rest of 150 m was used for test analysis.

The yarn tension was measured and equalized at the beginning of starching with a tensiometer tt. Schmidt model ETM. The concentration of the starch mass was measured continuously during the entire process with a Carl Zeiss refractometer, Jena, and kept constant by occasional (as needed) minimal dosing of a small amount of heated water into the reserve box, until the concentration in the box equalized.

### 2.4. Tensile Tester

The yarn tensile properties were tested according to ISO 2062, on a tensile tester tt. Mesdan, AutoDyn Strength Tester, and include the following properties: breaking force F (cN), breaking elongation ε (%) and tensile strength σ (cN/tex).

### 2.5. Abrasion Resistance Tester

Testing of yarn abrasion resistance was performed on a Zweigle Abrasion Tester G551, Zweigle, Reutlingen, Germany. The test is carried out simultaneously on 20 threads, under a load of 20 g. The roller over which the threads pass is covered with sandpaper (fineness 400) and moves left and right and rotates around its axis. The device is equipped with a counter that registers the number of movements of the roller. During the movement of the roller, the threads weaken and when the mass of the weight suspended on the thread exceeds the yarn strength, a break occurs, when the final number of roller movements is registered.

## 3. Results

**Figure 5:**
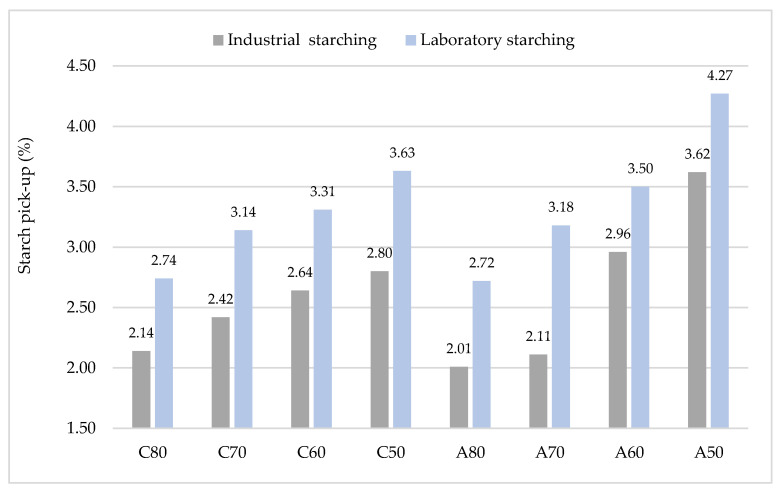
Starch pick-up on yarns after industrial and laboratory starching.

As it has already been pointed out, weaving preparation is an important process in the production of woven fabric that requires the yarn to starch before weaving for extra strength and abrasion resistance. During starching, a thin layer is formed (starch pick-up) on the surface to increase the strength of yarn and abrasion resistance whit reducing the yarn hairiness. Analyses have shown (Figure 5) that higher amounts of starch pick-up are retained on coarser yarns. By increasing the yarn fineness, the amount of starch pick-up decreases. In general, larger amounts of starch pick-up were achieved during laboratory starching.

In addition to the formed thin layer of starch on the yarn surface, a chemical bond is formed between starch molecules and fibres respectively crosslinking occurs. In a starch molecule, every glucose unit contains three hydroxyl groups, which provide adhesive capacity to fibres. These available hydroxyl groups of starch molecules are very reactive to form hydrogen bonds, to be oxidized and reduced, to form esters and ethers, or to be substituted by other functional groups [32]. During the starching process, the area of crystallinity in the fibre decreases, resulting in an increase in the amorphous area where the starch penetrates more easily than it does the crystals. That means that crosslinks are more easily produced between the macromolecules in the amorphous regions. The consequence is an increasing number of accessible reactive hydroxyl groups, and better bonding of the structural elements in the fibre, i.e., an increase in the number of crosslinks due to more intense entanglement and linking of polymer chains in amorphous areas. The crosslinking of starch chains with the chains of the structural elements in the fibres was confirmed by the obtained results of breaking force and abrasion resistance.

Results of breaking forces, elongation and strength before the starching process, and after industrial and laboratory starching processes are shown in Table 4.

Cotton yarns (label C) before starching, as well as aramid yarns (label A) show a change in breaking forces with a change in yarn fineness. The trend of increasing breaking forces follows a decrease in yarn fineness, that is, the coarser the yarn, the higher the breaking force. Breaking elongation in principle increases with increasing breaking forces for both cotton and aramid yarns.

Industrial starching increased yarn breaking forces and strength, which is the main goal of starching. The elongation at break in most samples decreased in cotton yarns, which represents the negativity of starching. This makes the yarn stiffer and less elastic, so the formation of the shed in the weaving becomes more difficult. Aramid yarns after industrial starching preserved higher breaking forces and strength, while breaking elongation decreased on finer yarns and even increased on coarser yarns, which represents a positive phenomenon. The greater difference in breaking elongations between cotton and aramid yarns was also transferred after starching.

By laboratory starching, the yarn gained more breaking forces and strength than by industrial starching. The shorter path of the starching process on a laboratory device (1:5), i.e., the shorter path of the wet yarn (when it is the most sensitive) did not experience stretching as in an industrial plant. The lower speed of the starching process (3 m/min in laboratory starching: 60 m/min in industrial starching) influenced more efficient laboratory starching in all cotton and aramid yarn samples.

**Figure 6:**
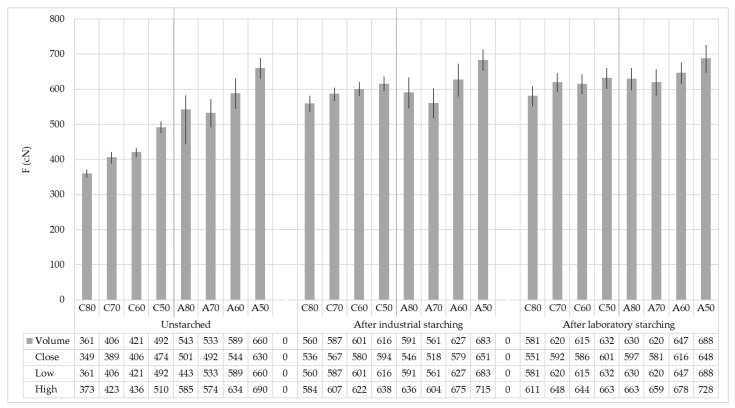
Yarn breaking forces before and after industrial and laboratory starching on samples with error intervals.

Figure 6 gives a clear overview of the yarn breaking forces before and after industrial and laboratory starching by samples, and the error intervals. Yarn fineness affected breaking forces and starching efficiency. Observing the overall results, it can be concluded that the coarser the yarn, the higher the breaking forces before and after starching. By starching, the finer yarn gained more breaking forces.

**Figure 7:**
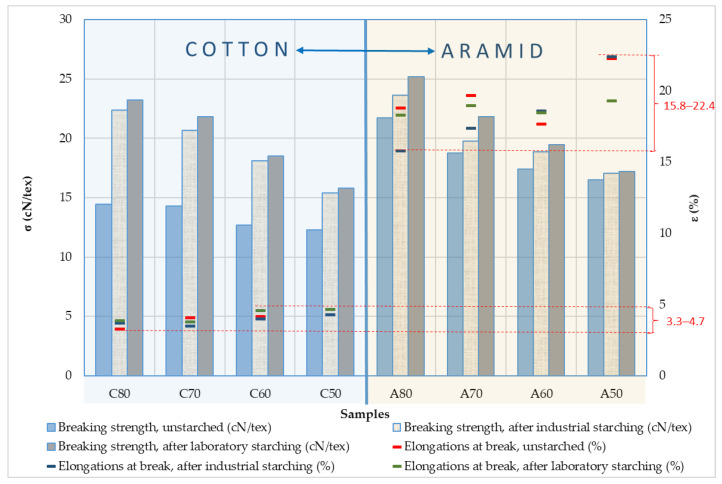
Breaking strength and elongation at break of cotton and aramid yarns before and after industrial and laboratory starching.

Based on the graphic representation in Figure 7, where the breaking strength and elongation at break of cotton and aramid yarns are shown, it can be concluded that the strength of the cotton yarns in the samples increased significantly by starching and that they are slightly higher after laboratory starching. By starching aramid yarns, the breaking strength increased significantly in finer yarns, especially yarn fineness 16.6 × 2 tex. Laboratory starching showed better results. The breaking elongation of cotton yarns before starching ranges from 3.3% (C_80_) to 4.7% (C_50_), after industrial starching from 3.7% (C_80_) to 4.3% (C_50_) and after laboratory starching from 3.8% (C_70_) to 4.7% (C_50_). Aramid yarns have a significantly higher elongation at break and with larger deviations between samples, so it amounts before starching from 17.7% (A_60_) to 22.3% (A_50_), after industrial starching 15.8% (A_80_) to 22.4% (A_50_) and after laboratory starching 18.3% (A_80_) to 19.3% (A_50_).

**Figure 8:**
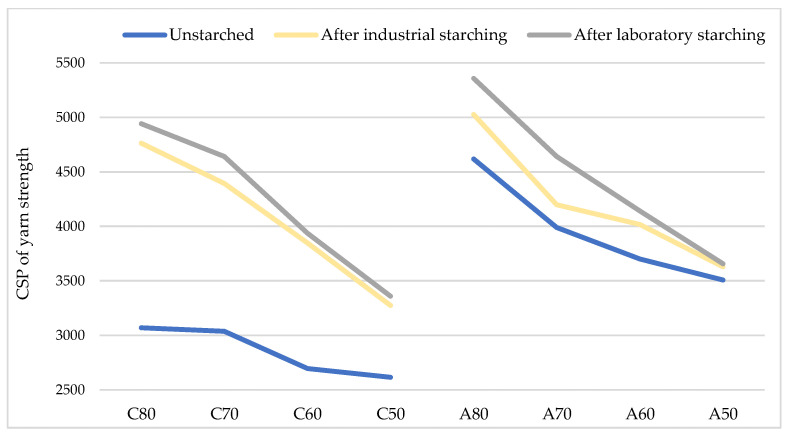
CSP of cotton and aramid yarns before and after industrial and laboratory starching.

Figure 8 shows the CSP values of cotton and aramid yarns before and after industrial and laboratory starching. CSP of unstarched cotton yarns ranged from 2615 to 3070, while for aramid yarns ranged from 3508 to 4618, which is an indicator of very high strength of the tested yarns. By increasing yarn fineness, CSP also increases. Starching increases yarn CSP, especially laboratory starching, and it is more significant for cotton yarns (in average for 45%) than for aramid yarns (in average for 10%).

**Figure 9:**
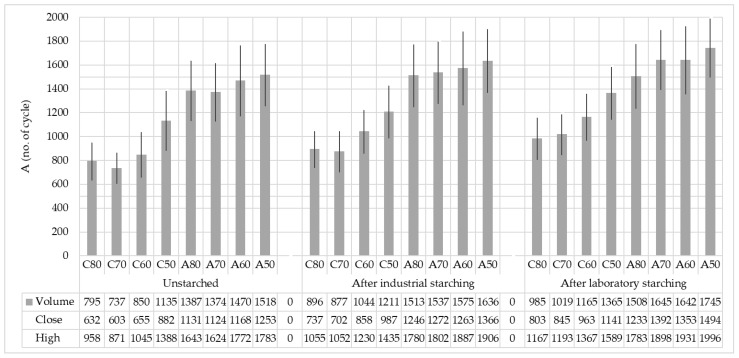
Abrasion resistance—number of cycles till break of cotton and aramid yarns before and after industrial and laboratory starching.

Table 5 and Figure 9 show the dependence of the influence of the starching process on abrasion resistance properties. In cotton samples, there is a visible trend of increasing abrasion resistance from finer yarns to coarser yarns in unstarched yarns and continues after the starching process has been carried out. The regular growth trend is not visible in aramid yarns as in cotton yarns, especially after starching. Intervals of error are slightly reduced by starching, and they are the smallest after laboratory starching. The error intervals show overlapping values between the samples in the groups, which indicates their insignificant difference.

Industrial and laboratory starching of the yarn resulted in an increase in the breaking forces, strength and abrasion resistance of all samples yarns (Table 6). Increases in breaking forces due to industrial starching are smaller compared to increases due to laboratory starching, ranging from 23 cN or 3.37% (A50) to 199 cN or 35.54% (C80). The increase in yarn breaking forces due to laboratory starching ranges from 28 cN or 4.07% (A50) to 220 cN or 37.87% (C80). If the difference in breaking force increase between starched cotton and aramid yarns is observed, a significant difference in favour of cotton yarn is visible. The increase in breaking forces due to industrial starching for cotton yarn ranges from 124 cN or 20.13% (C50) to 199 cN or 35.54% (C80), while for aramid yarn it ranges from 23 cN or 3.37% (A50) to 48 cN or 8.12% (A80). The increase in breaking forces due to laboratory starching shows a greater increase, so that for cotton yarns it ranges from 140 cN or 22.15% (C50) to 220 cN or 37.87% (C80), while for aramid yarns the values range from 28 cN or 4.07% (A50) to 87 cN or 14.03% (A80).

The breaking strength parameter also shows an increase in all tested starched yarns (Table 6). A greater increase in cotton yarn is evident, especially due to industrial starching, which ranges from 3.1 cN/tex or 25.20% (C50) to 7.96 cN/tex or 55.12% (C80), while in aramid yarn it ranges from 0.57 cN/tex or 3.45% (A50) to 1.92 cN/tex or 8.84% (A80). The increase in the strength of cotton yarn due to laboratory starching is also greater and amounts from 3.5 cN/tex or 28.46% (C50) to 8.80 cN/tex or 60.94% (C80), while in the case of aramid yarn, this increase is less and amounts from 0.7 cN/tex or 4.24% (A50) to 3.48 cN/tex or 16.02% (A80). Generally, it can be concluded that cotton yarns have a greater increase in strength than aramid yarns and that in both yarn groups, finer yarns gained more strength than coarser ones.

The yarn abrasion resistance also increased by starching (Table 6). Industrial starching resulted in a smaller increase of abrasion resistance, from 43 cycles or 4.12% (C60) to 101 cycles or 11.27% (C70) for cotton yarns, while for aramid yarns it ranges from 88 cycles or 5.48% (A50) to 145 cycles or 9.46% (A80). Laboratory starching resulted, as with the breaking forces, in a greater increase in cotton yarn abrasion resistance, from 154 cycles or 17.28% (C80) to 230 cycles or 16.85% (C50), while for aramid yarns, it ranges from 121 cycles or 8.02% (A80) to 271 cycles or 16.47% (A70).

**Figure 10:**
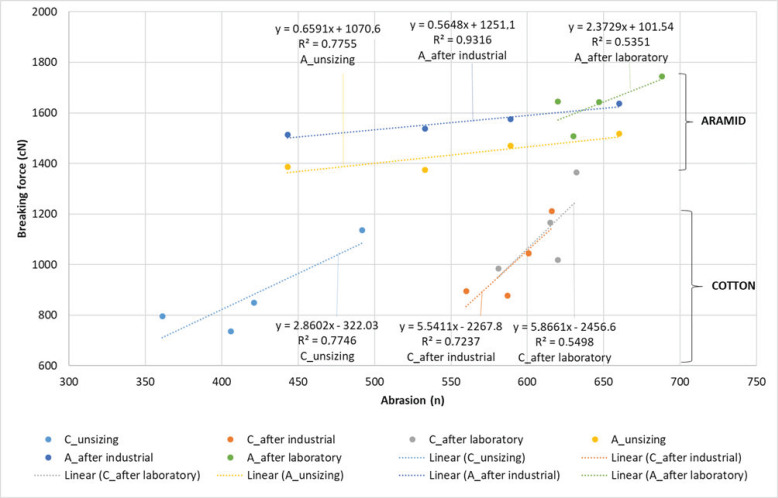
Regression lines, equation regression lines and correlation coefficient (R2) between breaking forces and abrasion resistance before and after industrial and laboratory starching.

The relationship between the yarn breaking forces before and after industrial and laboratory starching and the abrasion resistance is shown by regression lines with associated equations and correlation coefficients (Figure 10). According to the obtained analysis, it can be determined that cotton and aramid yarns have a relatively high degree of correlation between the breaking force values and abrasion resistance. Unstarched cotton yarns have a correlation coefficient of R^2^ = 0.7746, yarns starched by an industrial process have R^2^ = 0.7237, and yarns starched by a laboratory process have R^2^ = 0.5498. Unstarched aramid yarns have a correlation coefficient R^2^ = 0.7755, yarns starched by an industrial process R^2^ = 0.9316, and yarns starched by a laboratory process R^2^ = 0.5351. Laboratory starching of cotton and aramid yarns has lower correlation values of breaking forces and abrasion due to the influence of greater variations caused by time and length shorter starching process in laboratory conditions.

**Figure 11:**
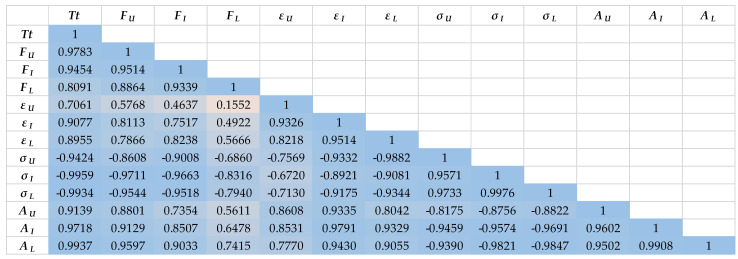
The correlation coefficient of parameters of cotton yarns before and after industrial and laboratory starching.

**Figure 12:**
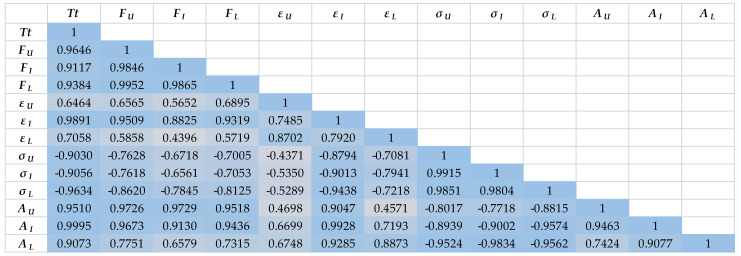
The correlation coefficient of parameters of aramid yarns before and after industrial and laboratory starching.

In general, by observing the correlation coefficients of the investigated parameters (Figure 11 and Figure 12), it is clear that the dependence between the parameters is weaker in the case of aramid yarns than in the case of cotton yarns. This represents an additional challenge when optimizing and implementing the entire starching process of aramid yarns, with the aim of eliminating defects and achieving more successful and efficient production, and thus higher quality of the finished fabric.

Furthermore, the parameters of both groups of tested yarns, measured after the implementation of the laboratory starching process, show a weaker mutual dependence, which is also understandable considering the laboratory conditions during the process implementation, which nevertheless affect the entire process differently than during the implementation of the industrial process. Thus, in the case of cotton yarns, the breaking force parameters of laboratory starched yarns correlate the weakest with all other tested parameters, as well as parameters of the elongation at break of non-starched yarns. In the case of aramid yarns, the breaking force parameters of laboratory and then industrially starched yarns have the weakest correlation with all other tested parameters, as well as the elongation at break of unstarched and laboratory starched yarns.

What also confirms the previously obtained results is the extremely strong dependence of the yarn fineness parameter with all the tested parameters. However, it can be also noted that the dependence is slightly weaker in correlation with elongation at break during laboratory starching.

## 4. Conclusions

Based on the conducted tests, detailed analyses and comparisons of cotton and aramid yarns of the same fineness were obtained. It can be concluded that the properties of cotton and aramid yarns are improved after the starching process, especially yarns of higher fineness.

Breaking forces of unstarched aramid yarns are higher than cotton yarns of the same fineness; starched samples follow the same trend. Increasing the fineness of the unstarched yarn reduces the yarns breaking force; the starched samples follow the same trend. The maximum increase in breaking forces was achieved on the finest cotton (35.54%/C80) and aramid (8.12%/A80) yarns starched by the industrial starching process, while the smallest increase was recorded on the coarsest yarns. The maximum increase in the breaking forces of yarns starched by the laboratory process shows the same trend as the increase in yarns starched by the industrial process, but to a slightly greater extent; for cotton yarns 37.87%/C80 and aramid yarns 14.03%/A80. This means that the yarns starched in laboratory conditions had smaller elongations and thus smaller deformations that occur when the yarn is in a wet state.

The yarns breaking elongation property was not significantly reduced by the starching process, especially during the laboratory starching of aramid yarns, which have a much wider range of value dispersion (15.8–22.4%) than cotton yarns (3.3–4.7%). For all starched yarn samples, the breaking strength also shows an increase. The maximum increase was achieved with cotton yarns (55.12%/C80), while aramid yarns achieve a significantly lower maximum value (8.84%/A80).

Cotton yarns after industrial (11.27%/C70) and laboratory (19.29/C70) starching provided higher abrasion resistance than aramid yarns starched by industrial (9.46%/A80) and laboratory (16.47/A70) procedure. By putting the yarn breaking forces and abrasion resistance in dependence, information is obtained about a relatively high degree of correlation (cotton: R^2^ = 9.7746, 0.7237, 0.5498, aramid: R^2^ = 0.7755, 0.9316, 0.5351), shown by regression lines with associated equations and correlation coefficients.

Ultimately, it can be concluded that the obtained values of breaking forces, breaking elongations, strength and abrasion resistance of yarns after the industrial and laboratory starching process give positive results, from which the justification of starching cotton and aramid yarns is confirmed. The obtained analyses and conclusions can serve as a basis for scientific research into the starching process of new materials and a procedure for properly optimizing the starching process. Laboratory starching is extremely important for the introduction of new starching procedures, and the development of new starching agents with new materials, to achieve the most economical and high-quality results in industrial production processes.

Due to their smoothness and uniformity, aramid fibres have a circular cross-section and provide a denser and more uniform yarn with a higher proportion of fibres in the cross-section than cotton fibres. This difference between cotton and aramid yarns is reduced if filament aramid fibres are cut and spun like cotton. Aramid spun yarn contains a smaller proportion of air space, so the penetration of the starch mass is more difficult, as well as its retention and binding in the yarn, which defines the properties of hydrophilicity and hydrophobicity less compared to cotton yarn. Aramid yarn, therefore, has a significantly higher strength than cotton yarn, but due to the surface protection, smoothing and elimination of static electricity, it is necessary to implement the starching process, which was confirmed by this research.

## Figures and Tables

**Table 1:** Tested samples.

Sample	Yarn Fineness		Raw Material Composition	Twist T (twist/m)	Unevenness U (%)
	Tex	Nm			
C_80_	12.5 × 2	80/2	100% Cotton	740 Z; 600 S	7.80
C_70_	14.2 × 2	70/2	100% Cotton	840 Z; 660 S	7.07
C_60_	16.6 × 2	60/2	100% Cotton	940 Z; 710 S	7.80
C_50_	20.0 × 2	50/2	100% Cotton	1030 Z; 760 S	8.45
A_80_	12.5 × 2	80/2	95.5% meta-aramid/ 5% para-aramid	970 Z; 670 S	4.80
A_70_	14.2 × 2	70/2	95.5% meta-aramid/ 5% para-aramid	1000 Z; 720 S	8.60
A_60_	16.6 × 2	60/2	95.5% meta-aramid/ 5% para-aramid	1170 Z; 800 S	7.60
A_50_	20.0 × 2	50/2	95.5% meta-aramid/ 5% para-aramid	1200 Z; 860 S	4.40

**Table 2:** Starching agents used in the starching process.

Starching Agents	Chemical Description	Industrial Values		Laboratory Values	
		l/kg	%	l/kg	%
Water (l)		500	88.03	5	88.03
Fibrosint C75 (kg)	Acrylic polymer modified polysaccharides	50	8.80	0.50	8.80
Inex 773C (kg)	Polyvinyl alcohol	15	2.64	0.15	2.64
Avirol 308AS (kg)	Compound based on fatty acid esters	3	0.53	0.03	0.53
Starch mass (l)		568	100	5.68	100

**Table 3:** Conditions of the starching process.

Condition	Value
Thread tension between creel for cross wound bobbins and starching trough	40 cN
Temperature of the starch mass in the starch box	75–80 °C
Starching speed	3 m/min
Pressure on the last pair of rollers for squeezing out the starch mass	19.1 N/cm^2^
Temperature on the cylinders of the contact dryer	140 °C
Starch mass concentration	7%

**Table 4:** Results of breaking forces, elongation at break and strength before the starching process, after industrial and laboratory starching processes.

Sample	Breaking Force		Elongation at Break		Breaking Strength	
	F (cN)	CV (%)	ε (%)	CV (%)	σ (cN/tex)	CV (%)
Before starching						
C_80_	361	3.45	3.3	5.32	14.44	3.451
C_70_	406	4.31	4.1	5.19	14.30	4.309
C_60_	421	3.53	4.2	4.87	12.68	3.528
C_50_	492	3.63	4.7	5.91	12.30	3.632
A_80_	543	7.72	18.8	6.78	21.72	7.724
A_70_	533	7.68	19.7	5.99	18.77	7.680
A_60_	589	7.66	17.7	7.27	17.40	7.661
A_50_	660	4.62	22.3	6.55	16.50	4.621
After industrial straching						
C_80_	560	4.31	3.7	7.33	22.40	4.31
C_70_	587	3.45	3.5	6.75	20.67	3.45
C_60_	601	3.53	4.0	6.51	18.10	3.53
C_50_	616	3.63	4.3	6.20	15.40	3.63
A_80_	591	7.72	15.8	15.73	23.64	7.72
A_70_	561	7.68	17.4	18.75	19.75	7.68
A_60_	627	7.66	18.6	13.70	18.89	7.66
A_50_	683	4.26	22.4	8.62	17.07	4.26
After laboratory straching						
C_80_	581	5.22	3.9	8.4	23.24	5.22
C_70_	620	4.59	3.8	8.2	21.83	4.10
C_60_	615	4.72	4.6	7.6	18.52	4.33
C_50_	632	4.92	4.7	7.1	15.80	3.82
A_80_	630	5.20	18.3	25.1	25.20	6.12
A_70_	620	6.27	19.0	10.6	21.83	6.21
A_60_	647	4.81	18.5	10.9	19.48	6.10
A_50_	688	5.88	19.3	11.1	17.20	6.62

**Table 5:** Abrasion resistance (A) of cotton and aramid yarns before and after industrial and laboratory starching.

Sample	A (no. of Cycles)					
	Before Starching		After Industrial Starching		After Laboratory Starching	
	$\overset{-}{X}$	CV (%)	$\overset{-}{X}$	CV (%)	$\overset{-}{X}$	CV (%)
C_80_	795	22.14	896	19.42	985	20.42
C_70_	737	16.83	817	19.55	891	17.67
C_60_	1001	19.46	1044	17.84	1165	17.34
C_50_	1135	22.31	1211	18.53	1365	16.40
A_80_	1387	18.45	1533	17.43	1508	18.24
A_70_	1374	18.22	1477	17.94	1645	15.39
A_60_	1470	20.52	1575	19.83	1672	17.30
A_50_	1518	17.44	1606	16.83	1645	15.26

**Table 6:** Absolute and relative values of increase in breaking forces (cN, %) and abrasion resistance (no. of cycle, %) of yarns after industrial and laboratory starching.

Sample	I_FI_ (cN)	I_FL_ (cN)	Iσ_I_(cN/tex)	Iσ_L_ (cN/tex)	I_AI_ (no. of Cycle)	I_AL_ (no. of Cycle)	I_FI_ (%)	I_FL_ (%)	Iσ_I_ (%)	Iσ_L_ (%)	I_AI_ (%)	I_AL_ (%)
C_80_	199	220	7.96	8.80	80	154	35.54	37.87	55.12	60.94	9.79	17.28
C_70_	181	214	6.38	7.54	101	190	30.83	34.52	44.65	52.76	11.27	19.29
C_60_	180	194	5.42	5.84	43	165	29.95	31.54	42.74	46.06	4.12	14.16
C_50_	124	140	3.10	3.50	76	230	20.13	22.15	25.20	28.46	6.28	16.85
A_80_	48	87	1.92	3.48	145	121	8.12	13.81	8.84	16.02	9.46	8.02
A_70_	28	87	0.98	3.06	103	271	4.99	14.03	5.22	16.30	6.97	16.47
A_60_	38	58	1.49	2.08	105	202	6.06	8.96	8.56	11.95	6.67	12.08
A_50_	23	28	0.57	0.70	88	127	3.37	4.07	3.45	4.24	5.48	7.72

where: I_FI_—increasing breaking force by industrial starching (cN, %), I_FL_—increasing breaking force by industrial starching (cN, %), Iσ_I_—increasing breaking strength by industrial starching (cN/tex, %), Iσ_L_—increasing breaking strength by laboratory starching (cN/tex, %), I_AI_—increasing abrasion resistance by industrial starching (no. of cycle, %), I_AL_—increasing abrasion resistance by laboratory starching (no. of cycle, %).

## Data Availability

The data presented in this study are available on request from the corresponding author. The data are not publicly available due to the preparation of a doctoral thesis.
